# A regression for estimating metabolizable glucose in diets of weaned piglets for optimal growth performance

**DOI:** 10.5713/ab.20.0459

**Published:** 2020-12-11

**Authors:** Liangkang Lv, Zhi Feng, Dandan Zhang, Long Lei, Hui Zhang, Zhengya Liu, Ying Ren, Shengjun Zhao

**Affiliations:** 1 Hubei Key Laboratory of Animal Nutrition and Feed Science, Wuhan Polytechnic University, Wuhan 430023, China

**Keywords:** Blood Vessels Cannulation, Feed Evaluation, Ileal Fistula, Metabolizable Glucose, Weaned Pigs

## Abstract

**Objective:**

Two experiments were conducted to provide a new approach for evaluating feed nutritional value by metabolizable glucose (MG) in piglet diets with different levels of starch and crude fiber. In Exp 1, a regression equation for MG was generated. In Exp 2, the equation was verified, and the optimal growth performance of piglets under appropriate MG levels was tested.

**Methods:**

In Exp 1, 20 weaned piglets (7.74±0.81 kg body weight [BW]) were randomly assigned to 1 of 4 treatments, including the basal diet containing different levels of MG (starch, 25.80%, 31.67%, 45.71%, 49.36%; crude fiber, 1.23%, 1.35%, 1.80%, 1.51%). The piglets were implanted with an ileal fistula, cannulation of the carotid artery, portal vein, and mesenteric artery. The chyme from the ileum fistula and blood samples were collected. In Exp 2, 30 weaned piglets (8.96±0.50 kg BW) were randomly assigned to 1 of 5 treatments, including the experimental diets with different levels of MG (37.6, 132.5, 300.0, 354.3, and 412.5 g/kg). The piglets’ BW, and feed consumption were recorded to calculate growth performance during the 28-d experiment.

**Results:**

In Exp 1, the MG levels in 4 diets were 239.62, 280.68, 400.79, and 454.35 g/kg. The regression equation for the MG levels and dietary nutrients was: Y (MG) = 12.13×X_1_ (starch)+23.18×X_2_ (crude fiber)−196.44 (R^2^ = 0.9989, p = 0.033). In Exp 2, treatments with 132.5 and 300.0 g/kg MG significantly (p<0.05) increased average daily gain and feed conversion efficiency of weaned piglets, increased digestibility of crude fat, and had no effect on digestibility of crude protein compared to 300.0 to 412.5 g/kg MG.

**Conclusion:**

The pig model combining the ileum fistula and cannulation of blood vessels was successfully used to determine the dietary MG levels. The recommended MG level in weaned pig diets is 132.5 to 300.0 g/kg.

## INTRODUCTION

Glucose, a highly absorbable dietary monosaccharide, is oxidized for energy through glycolysis and the Krebs cycle [1,2]. Previous studies have shown that the level of carbohydrates in the diet is one of the key factors affecting gut development in weaned piglets, and that the morphology of the small intestine and average daily gain (ADG) of piglets do not differ between dietary starch and glucose [3]. Moreover, dietary crude fiber was shown to affect large intestine fermentation and further change the production of volatile fatty acids [4], and the propionic acid and lactic acid provided from the large intestine were shown to be converted into glucose in the liver [5]. Therefore, a certain amount of glucose is available for animals during the metabolism process. Considering this available glucose, Lu [6,7] proposed the theory of metabolizable glucose (MG), in which one exogenous glucose is obtained by hydrolysis of starch in the digestive tract and one endogenous glucose obtained by gluconeogenesis.

To accurately measure the domestic livestock’s energy requirements, animal nutritionists started working on the role of MG in energy conversion and substance metabolism. However, the research on this subject is still rare [8–10]. In the meantime, the levels of carbohydrates, crude protein, and crude fat are the main energy evaluation indexes of the animal diet used in the current feed evaluation system. However, these indexes may not accurately represent the pig’s demand for nutrition due to neglecting the energy changes during substance metabolism [11,12]. Hence, some studies have focused on objectively refining the metabolizable energy for the evaluation of feed quality and energy, and the application of MG to ruminants [6–10]. In addition, volatile fatty acid absorbed in the large intestine was estimated to meet about 5% to 25% energy maintenance needs of pigs [13]. This means that the energy provided by endogenous MG is also crucial for the growth and development of monogastric animals.

In our previous studies, we have measured the exogenous MG in weaned piglets by installing an ileal fistula to evaluate the small-intestinal digestibility of starch [14]. Based on the results, we hypothesized that endogenous MG could be calculated by determining the absorption of propionic acid and lactic acid in the portal vein. Thus, we herein explored the appropriate MG levels to achieve optimal growth performance of weaned piglets, a new concept for feed evaluation of pigs based on MG. In general, the objectives of this study were i) to establish a regression equation for MG and different starch, crude fiber levels in weaned piglets’ diet. ii) to evaluate the effects of different dietary MG levels on growth performance and the digestibility of nutrients in weaned piglets.

## MATERIALS AND METHODS

### Animal care

The experimental procedures were approved by the Animal Care and Use Committee of Wuhan Polytechnic University, Hubei Province, China (approval number: 2010-0029).

### Animals, diets, and experimental design

In Exp 1, 20 healthy female crossbred (Duroc×Large White× Landrace) weaned piglets (7.74±0.81 kg of body weight [BW]; 25 d of age; early weaned at 21 d of age) were randomly assigned to four dietary treatments (on d 0). There were five pens per treatment with one pig per pen. The gradual weaning method was used to artificially reduce the contact between sows and piglets (isolation during the day [8:00 to 20:00], contact at night), the number of lactations, and the sows’ feed intake (from d −8 to d −4). During this period, a small amount of creep feed (approximately 120 g/d per pig; Wuhan ANYOU Feed Technology Co., Ltd, Hubei, China) was provided to the piglets. On the weaning day (d −4), all the sows were transferred from the farrowing house while the piglets were kept. The experimental diet ratio to the creep feed in the total piglet’s diet was gradually increased (from 0% to 100%) over the next 4 days (from d −4 to d 0), with *ad libitum* access to feed and clean water. The four experimental diets labeled I to IV (Table 1) were formulated with different levels of MG to meet the nutrient requirements for weaned piglets, according to the NRC [15]. The 16.7% edible corn starch (Jinan Mingqi Biological Technology Co., Ltd, Shandong, China) was used as the other starch source in Diet IV. Titanium dioxide (TiO_2_, 1 g/kg diet) was added to each diet as the indigestible marker to measure nutrient digestibility.

During a 7-day adaptation period (d 1 to d 7), the piglets had free access to water and were fed the experimental diet at 8:00, 12:00, 16:00, and 20:00 (75 g feed/meal). All piglets were anesthetized with isoflurane and placed in left lateral recumbency. The abdominal cavity was opened, and T-type cannula fistulas were installed in the piglets’ terminal ileum [16]. By separating the connective tissues from the portal vein around the targeted site, cannulation (0.62 mm ID and 1.02±0.03 mm OD, Instech Laboratories, Inc., Plymouth Meeting, PA, USA) was inserted into the portal vein for approximately 4 to 5 cm, toward the hilum of the liver. The end of the cannulation was connected with the polyurethane external infusion tubing (Instech Laboratories, Inc., USA) outside of the body. According to the same surgical procedure, mesenteric vein cannulation and carotid artery cannulation (0.31 mm ID and 0.64±0.05 mm OD, Instech Laboratories, Inc., USA) were installed [17]. The operation wound was disinfected three times a day, and cannulation was flushed with saline and sterile heparin sodium (200 IU heparin/mL of 0.9% NaCl solution) daily to maintain patency. The piglets had recovered and resumed normal feed intake 3 to 5 days after the operation, after which they were fasted for 12 h. On d 15, based on the one twenty-fourth of average daily feed intake (ADFI), feeding was provided every hour to the pigs (12.5 g/h; from 6:00 to 15:00). Meanwhile, 1% P-aminohippuric acid (PAH) was continuously infused into the mesenteric vein via a screw-driven syringe constant-infusion pump. The infusion rate was started at 3.820 mL/min for 5 min and then changed to 0.788 mL/min for the next 7 h (8:00 to 15:00) [18].

In Exp 2, 30 healthy female crossbred (Duroc×Large White×Landrace) weaned piglets (8.96±0.50 kg of BW on 35 d of age; early weaned at 21 d of age) were randomly assigned to five dietary treatments in a completely randomized design for a 28-d experiment, with *ad libitum* access to feed and water. Each treatment diet was fed to pigs in six replicated pens with one pig per pen. The five diets (Table 2) were formulated to meet or exceed the nutrient requirements recommended by NRC (2012) [15]. According to the regression equation of Exp 1, diets consisted of the following five levels of MG: 37.6 g/kg (Diet A), 132.5 g/kg (Diet B), 300.0 g/kg (Diet C), 354.3 g/kg (Diet D), and 412.5 g/kg (Diet E).

### Sample collection

In Exp 1, on d 15, blood samples were collected from both the portal vein and carotid artery at 8:00 (baseline blood; before perfusion), 9:00, 10:00, 11:00, 12:00, 13:00, 14:00, and 15:00, placed into heparinized vacuum tubes, centrifuged at 2,550×*g* for 15 min at 4°C, and then stored at −80°C for analysis. In the meantime, ileal digesta was collected from a T-type cannula fistula. The clean rubber balloon was placed on the opened cannula fistula. Digesta flow was initially intermittent, and balloons were replaced approximately every 30 min until the digesta began to flow consistently. Samples were immediately placed on ice and refrigerated at −20°C before further analysis. This procedure was followed to reduce the possibility of bacterial proliferation and fermentation in the balloons.

In Exp 2, all pigs were weighed on a platform, at the beginning (on d 1) and on the last day (on d 28) of the test, and feed consumption was recorded daily to calculate the ADG, ADFI, and feed conversion efficiency (FCE). Fecal samples were collected from each pig for three days (from d 26 to 28) and were stored at −20°C. After thawing, the three-day collection of fecal samples was pooled, and then dried (at 68°C for 48 h) for further analysis.

### Plasma organic acid concentrations

The quantification of propionic acid in plasma was carried out by gas chromatography, which was entrusted in the Feed Efficiency and Safety Supervision and Testing Center of the Ministry of Agriculture (Beijing, China). The lactic acid concentrations in the blood samples were analyzed using a LA (HY-N0056) KIT according to the manufacturer’s instructions (Beijing Sino-UK Institute of Biological Technology, Beijing, China).

### Portal vein-blood flow rate and organic acid net absorption

PAH concentrations in plasma were analyzed as described in previously published literature [19]. The portal blood flow rate (F, L/kg/h) was calculated using the formula: F = Ci×IR× ([PAH_p_−PAH_a_]×BW)^−1^ [19], where Ci refers to the injected concentration of PAH into the mesenteric vein, in mg/L; IR refers to the injected rate of PAH, in mg/min; PAH_p_ and PAH_a_, respectively refer to the concentrations of PAH in the mesenteric vein and carotid plasma; and BW refers to the body weight, in kg. The organic acid net absorption in the portal vein (F_OA_, g/d) was calculated using the formula: F_OA_ = (C_p_−C_a_)×F [20], where C_p_ and C_a_ refer to the concentrations of organic acids, in μg/mL, measured in the blood of the portal vein and carotid artery, respectively.

### Starch content and digestibility of nutrients

Starch content was determined using enzymatic methods [21]. The digestibility of nutrients (Exp 1 and Exp 2) was measured following a previously published procedure [22]. The levels of nutrients in the feed (NR), digesta (NF) samples, and the concentration of TiO_2_ in the feed (IR) and digesta (IF) samples were determined. The digestibility of nutrients (D) was calculated using the following equation: D = (1−IR×NF/IF×NR)×100%. Notice that the digestibility of nutrients is measured based on nutrient levels in small intestine digesta in Exp 1 but in the feces in Exp 2.

### Intake of metabolizable glucose

The intake of MG was calculated by the formula: MG = IDSG+LAFG = K_1_×S×0.9+0.83×K_2_×Pr+0.87×K_3_×La [7], where IDSG refers to exogenous MG; LAFG refers to endogenous MG; S refers to the starch contents in the small intestine; Pr and La refer to propionic acid and lactic acid content by the fermentation of microflora in the large intestine, respectively; K_1_ refers to the digestibility of starch in the small intestine; K_2_ refers to the absorptivity of propionic acid in the large intestine; K_3_ refers to the absorptivity of lactic acid in the large intestine; 0.9, 0.83, and 0.87 are the conversion coefficients of starch, propionic acid, and lactic acid into glucose, respectively.

### Statistical analysis

Data for both experiments were analyzed by one-way analysis of variance test using the general linear model procedures of SPSS version 20.0 (SPSS Inc., Chicago, IL, USA). Post hoc testing was performed using Duncan’s multiple comparison tests. Data were expressed as mean±standard deviation, and differences were considered significant at p-value <0.05. In Exp 1, the relationship between MG and the nutrients in the feed was measured using linear regression analysis procedures of SPSS.

## RESULTS

### The small intestine digestibility of starch and dry matter in Exp 1

The four diets’ starch content was 25.80%, 31.67%, 45.71%, 49.36%, respectively, and there were significant differences (p<0.05, in Table 3). Piglets fed Diet IV had higher starch content in digesta than piglets fed Diet I (p<0.05). No significant difference was found in the TiO2 content of diets and small intestine digesta (p>0.05). Compared with piglets fed Diet IV, piglets fed with Diet II had a higher small intestine digestibility of dry matter (p<0.05). The digestibility of starch in the small intestine increased with the starch content of diet in the four groups, and piglets fed high starch diets of 45.71% and 49.36% had higher starch digestibilities of 89.89% and 90.32% compared with piglets fed starch diets of 25.80% and 31.67% (p<0.05).

### Portal vein net absorption of lactic and propionic acids in Exp 1

The portal blood flow rate (Table 4) of piglets under the four diets were 4.85, 9.98, 6.32, and 8.18 L/kg/h, piglets fed Diet II showed greater portal blood flow rate than piglets fed Diet I and III (p<0.05), and piglets fed Diet IV had greater portal blood flow rate than piglets fed Diet I (p<0.05). The propionic acid concentrations of the carotid artery plasma were undetectable by gas chromatography. No significant difference was found in the propionic acid concentrations of portal plasma among Treatments (p>0.05). Relative to Diet III, piglets fed Diet I had higher lactic acid concentrations in the carotid artery plasma (p<0.05). Diet I supplementation resulted in a significant decrease in the lactic acid concentrations of portal plasma compared to Diet II, III, and IV (p< 0.05). A significant increase in propionic acid net absorption of the portal vein was observed in Diet II pigs, compared to Diet I and IV piglets (p<0.05). Piglets fed Diet I had higher lactic acid net absorption of the portal vein than piglets fed Diet III and IV (p<0.05).

### Diet metabolizable glucose levels for weaned piglets in Exp 1

The exogenous MG intake in piglets increased with increasing starch content in the diet, and there was a significant difference among the four treatments (p<0.05; Table 5). A positive correlation was found between the exogenous MG levels, exogenous MG intake and starch levels in diets (p< 0.05). There was no significant difference in the endogenous MG intake and endogenous MG levels in diets among treatments (p>0.05). In general, the MG intake was increased with increasing starch level, and piglets fed Diet III and IV had higher MG intakes and MG levels in the diet compared to piglets fed Diet I and II (p<0.05).

### Correlation of metabolizable glucose in diets and feedstuffs in Exp. 1

As shown in Table 6, the regression equation of the exogenous MG levels in diet and starch, and crude fiber content of diet was: Y_1_ = 12.07×X_1_+5.80×X_2_−110.82 (R^2^ = 0.9995, p< 0.05). The regression equation of the endogenous MG levels in diet and starch, crude fiber content of diet was: Y_2_ = 2.35×X_1_ +18.48×X_2_−106.70 (R^2^ = 0.6298, p = 0.608). The regression equation of MG levels in diet and starch, crude fiber content of diet was: Y_3_ = 12.13×X_1_+23.18×X_2_−196.44 (R^2^ = 0.9989, p = 0.033).

### Growth performance and digestibility of nutrients in Exp 2

In Exp 2, there was no difference in ADFI among Treatment Diets B, C, and D (p>0.05; Table 7). The piglets fed Diet E had more ADFI than the piglets fed Diet A, B, and C (p<0.05), and the piglets fed Diet B and C showed higher ADFI than piglets fed Diet A (p<0.05). The results showed that the piglets fed Diet B and C had higher ADG and FCE compared with the piglets fed Diet A, D, and E (p<0.05), but the difference in ADG in Treatment Diet A and Diet D was not obvious (p>0.05), and piglets fed Diet E had greater ADG than piglets fed Diet A (p<0.05). Meanwhile, no differences in FCE were observed among Diets A, D, and E (p>0.05).

A significant elevation from 55.36% to 65.72% of digestibility of dry matter occurred when MG levels were increased from 37.6 to 132.5 g/kg (p<0.05), no differences in the digestibility of dry matter were found with the incremental levels of MG from 132.5 to 354.3 g/kg (p>0.05). The digestibility of dry matter was the highest in piglets fed Diet E at 412.5 g/kg MG (p<0.05). In addition, piglets fed Diet A at 37.6 g/kg MG showed the lowest digestibility of crude protein (p<0.05). No differences in digestibility of crude protein were found with the levels of MG from 132.5 to 412.5 g/kg (p>0.05). No significant effects were observed on the digestibility of crude fat in piglets fed diets of 37.6, 132.5, 300.0, and 412.5 g/kg MG (p>0.05), and piglets fed Diet D of 354.3 g/kg MG showed the lowest digestibility of crude fat compared to piglets fed diets from 37.6 to 300.0 g/kg MG (p<0.05).

## DISCUSSION

The previous study has shown that the heifers’ potential growth performance would be improved under the optimal balance between the amount of physically effective neutral detergent fiber to MG [8]. To date, there has been no research on MG in pigs. Thus, in Exp 1, the pig model was used for combining the ileum fistula and cannulation of blood vessels to measure exogenous MG and endogenous MG, respectively. As expected, no significant difference in TiO_2_ content in small intestine digesta was found among treatments, which indicates the reliability of using the TiO_2_ as an exogenous indicator to measure nutrient digestibility. The present study found that the small intestine digestibility of dry matter significantly increased in treatment Diet IV. This could be because glucose absorbed in the small intestine increased with increasing starch content, resulting in more glucose consumption, which fuels the small intestine in a positive way. Moreover, the piglets fed the diet with a higher starch level had the better small-intestinal digestibility of starch within an applicable range of values. To a large extent, that was because more starch provides a positive stimulation to produce more amylase, but excess of starch might result in the overproduction of glucose in the small intestine, reducing the release of digestive juices. Therefore, in our viewpoint, the different exogenous MG levels could affect the ability of the small intestine to digest and absorb.

The endogenous MG available to animals is determined by the portal blood flow rate, lactic acid, and propionic acid produced by the fermentation of large intestine microorganisms [6,7]. The results of Exp 1 showed that the portal blood flow rate was 4.85 to 9.98 L/h per kg BW. In contrast, the portal blood flow rates of 2.4 to 3.6 L/h per kg BW were reported by Anderson [23] with the same PAH infusion method in pigs weighing 27 kg. It has also been reported that the portal blood flow rate values for the pre- and post-prandial periods, respectively, were 1.89 and 2.48 L/h per kg BW, with five pigs averaging 37 kg [17]. Moreover, when all 13 pigs averaging 54 kg were considered, the portal blood flow rate values were 1.69 and 2.20 L/h per kg BW [17]. This implies that the portal blood flow rate might decrease with somatic growth, and the feeding regime might increase the portal blood flow rate. The differences between the reasons might be the BW of pigs, feeding regime (feeding every hour in this study), and dietary components. The propionic acid concentrations of the carotid artery plasma were undetectable by gas chromatography, which could be due to the fact that few propionic acids produced in the large intestine were completely metabolized by the liver. The study showed that the propionic acid concentrations of portal plasma were 4.29 to 5.18 μg/mL for growing pigs (BW = 33.3 kg) under different starch sources [24]. However, the propionic acid concentration of portal plasma for piglets (BW = 7.74 kg; in Exp 1) was 2.50 to 3.49 μg/mL. This discrepancy might be attributed to less feed intake, gut microbiome differences, and low capacity of the large intestine compared to that of growing pigs. Moreover, the different propionic acid and lactic acid net absorption of the portal vein among Treatments could be caused by diets with different diet components. The yield and composition ratio of fermentation end-products in the large intestine were influenced by microorganisms, and the levels of nutrients in the diet can directly affect the microbiota in the large intestine [25]. In addition, there is a pair of opposite effects. One is that more substrates (crude fiber) are provided for the fermentation of microflora in the large intestine. The other is that the emptying rate of the large intestine would be accelerated with the increase in crude fiber levels. It is worth noting that the mechanism behind the surging value of the portal flow rate and the propionic acid net absorption of the portal vein for pigs fed Diet II (280.68 g/kg MG, in Exp 1) remains unclear. Interestingly, this could correspond to the better growth performance of the piglets fed Diet B and C (132.5 to −300.0 g/kg MG, in Exp 2), which requires further studies. Based on the preceding, the results showed that the changing trend of portal blood flow rate and the lactic acid, propionic acid production in the large intestine was not consistent. Thereby, it reflected that the plain calculations might not gain endogenous MG intake for diet formula.

The evaluation system of metabolizable nutrients unifies the dietary nutrient level and the nutrient requirement of pigs, and the evaluation system of metabolizable nutrients could be more accurate and appropriate compared with the evaluation system of dietary nutrient levels and their digestibility [6,7]. There is a gradual refinement process by converting the utilization levels of nutrients into MG requirements for pigs. Moreover, the endogenous MG refers to the glucose transferred in the liver as propionic acid and lactic acid produced in the large intestine, but this part of the energy in propionic acid and lactic acid is overlooked in the existing evaluation system of feed nutrition value. However, our data demonstrated that endogenous MG was roughly a fifth of the exogenous MG. Previous studies have shown that absorbed volatile fatty acids in the large intestine could meet approximately 20% to 30% of the piglets’ maintenance energy needs [26], which could support for our results and further reflect the non-negligible for endogenous energy. Thus, to develop a new method of feed nutrition evaluation of MG, the MG levels in diets were calculated based on the feed intake (300 g/d), with the value of 239.62±28.22 g/kg, 280.68±17.52 g/kg, 400.79±5.38 g/kg, and 454.35±13.51 g/kg corresponding to the four experimental diets, respectively.

To evaluate the nutritional value of feed more intuitively *in vitro*, we tested the regression equations of MG and some nutrients (starch and crude fiber) in the diet. The data of Exp 1 showed that the single regression equation of MG and starch was: Y = 8.92×X_1_+3.699 (R^2^ = 0.991), the single regression equation of MG and crude fiber was: Y = 305.827×X_2_ −106.47 (R^2^ = 0.563), but the multiple regression equation of the MG and starch and crude fiber in feed was: Y = 12.13×X_1_ +23.18×X_2_−196.44 (R^2^ = 0.9989), with the MG level in diet as the dependent variable Y, and starch, crude fiber as independent variables X_1_ and X_2_. As expected, the multiple regression equation could be more appropriate for estimating the MG level in the diet. Note that the digestible starch in the diet is digested by amylase in the small intestine, but resistant starch can only be fermented by microflora to produce organic acids in the large intestine. In order to unify the two values, we chose the total starch content in this study. This may affect the accuracy of the separate regression formulas for exogenous MG and endogenous MG, but the MG regression equations should still be applicable.

Based on the linear regression equation obtained from this experiment, our researchers designed Exp 2 to explore the optimal MG level for piglet diet under isocaloric and isonitrogenous conditions. The results of Exp 2 indicated that dietary MG level did affect the growth performance of piglets, and the highest ADG and FCE were obtained when piglets were fed the diet of 132.5 and 300.0 g/kg MG. In the meantime, ADFI and digestibility of dry matter were increased as the incremental levels of MG in diets. The probable reason is that the palatability of piglets was improved by increasing the amount of corn starch. More dry matter intake had no effect on FCE, even though it might have caused the growth of weaned piglets [27], which was consistent with our results. In addition, the co-treatment group with both higher crude protein digestibility and crude fat digestibility was Treatment Diets B and C (132.5 and 300.0 g/kg MG). It could be due to the supply of glucose was reduced with less MG in diets, while higher levels of MG treatments might lead to the supersaturated state of glucose, thereby reducing the ability of digestion and absorption of nutrients [8]. The results of Exp 2 showed that piglets fed the diet of appropriate MG level (132.5 and 300.0 g/kg) could apparently enhance the digestibility of nutrients, thereby improving growth performance, which was consistent with the previous study [28]. Overall, Exp 2 suggests that the energy system may not be the only way to evaluate the feed, which also shows the importance and significance of studying the MG in pigs. However, the digestion and metabolism of pigs at different growth stages (e.g., weaned piglets vs. growing pigs) are diverse and complex, and it might be impossible to express the requirements of MG for full-stage pigs with one equation. The regression equation of MG in this study was only applied to weaned piglets, and more associated research on the optimal MG levels in the diet for different stages pigs is needed.

## CONCLUSION

In summary, we designed this pig’s model for the determination of MG in piglets and obtained the regression equation to estimate MG in piglet’s diet. Meanwhile, we recommended the dietary MG of 132.5 to 300.0 g/kg to achieve optimal growth performance of weaned piglets. This finding might provide the new thought for feed evaluation in piglets.

## Figures and Tables

**Table 1:** Ingredients and nutritional composition of experimental diets in Exp 1

Items	Treatment			

	Diet I	Diet II	Diet III	Diet IV
Ingredients (%, as fed basis)^1)^				
Corn	12.00	22.00	47.38	27.30
Corn starch	0.00	0.00	0.00	16.70
Wheat bran	16.00	12.00	2.45	2.00
Soya meal	10.00	10.00	10.00	10.00
Puffed soybean	10.00	10.00	10.00	10.00
Soybean oil	15.75	12.54	4.35	4.46
Corn gluten meal	9.10	7.53	3.52	6.58
Fish meal	5.00	5.00	5.00	5.00
Plasma protein powder	4.00	4.00	4.00	4.00
Whey powder	10.00	10.00	10.00	10.00
Stone powder	0.50	0.50	0.50	0.50
Calcium hydrogen phosphate	1.20	1.20	1.20	1.20
Salt	0.30	0.30	0.30	0.30
Zeolite powder	4.92	3.71	0.080	0.73
Methionine	0.23	0.22	0.22	0.23
Premix^2)^	1.00	1.00	1.00	1.00
Total	100.00	100.00	100.00	100.00
Nutritional composition				
Metabolic energy (MJ/kg)^3)^	13.35	13.35	13.35	13.35
Starch (%)^4)^	25.80	31.67	45.71	49.36
Crude fiber (%)^4)^	1.23	1.35	1.80	1.51
Crude protein (%)^3)^	21.19	21.19	21.19	21.19
Total calcium (%)^3)^	0.89	0.89	0.89	0.89
Total phosphorus (%)^3)^	0.70	0.72	0.77	0.73
Total lysine (%)^3)^	1.23	1.24	1.27	1.25
Total methionine (%)^3)^	0.60	0.59	0.59	0.60

1) Extra add titanium dioxide (1 g per kg diet) as the indigestible marker to measure nutrient digestibility [24].

2) The premix provided the following amounts per kilogram of complete diet: Vit A ll, 500 IU; Vit D_3_ 4,000 IU; Vit E 45 mg; Vit K_3_ 3.5 mg; Vit B_3_ 2 mg; Vit B_2_ 6.5 mg; Vit B_6_ 5 mg; Vit B_12_ 0.05 mg; niacin 25 mg; calcium pantothenate 9.5 mg; folic acid 0.65 mg; biotin 0.5 mg; choline chloride 500 mg; Vit C 288 mg; Fe l80 mg; Cu l40 mg; I 1 mg; Se 0.27 mg; Zn l 50 mg; Mg 68 mg; Co 0.3 mg.

3) Calculated.

4) Analyzed.

**Table 2:** Ingredients and nutritional composition of experimental diets in Exp 2

Items	Treatment				

	Diet A	Diet B	Diet C	Diet D	Diet E
Ingredient (%, as fed basis)					
Corn	0.00	5.54	16.10	34.60	19.30
Corn starch	0.00	0.00	0.00	0.00	19.00
Wheat bran	0.50	11.36	12.46	1.00	0.00
Rice chaff	5.21	2.99	2.22	3.18	3.80
Soya meal	12.01	11.09	10.39	10.00	10.00
Puffed soybean	12.01	11.09	10.39	10.00	10.00
Soybean oil	25.45	20.10	15.02	9.86	7.71
Corn gluten meal	15.30	10.40	7.46	7.05	9.55
Fish meal	4.80	4.44	4.15	4.00	4.00
Plasma protein powder	3.60	3.33	3.12	3.00	3.00
Whey powder	9.60	8.87	8.31	8.00	8.00
Stone powder	0.60	0.55	0.52	0.50	0.50
Calcium hydrogen phosphate	1.44	1.33	1.25	1.20	1.20
Salt	0.36	0.33	0.31	0.30	0.30
Zeolite powder	6.00	5.54	5.19	4.31	0.61
Lysine	0.24	0.22	0.21	0.20	0.20
Methionine	0.28	0.22	0.23	0.20	0.23
Titanium dioxide	0.10	0.10	0.10	0.10	0.10
Premix^1)^	2.50	2.50	2.50	2.50	2.50
Total	100	100	100	100	100
Nutritional composition					
Metabolizable glucose (g/kg)^2)^	37.60	132.50	300.00	354.30	412.50
Metabolic energy (MJ/kg)^2)^	13.67	13.67	13.68	13.66	13.65
Starch (%)^3)^	8.70	15.50	22.20	28.70	36.70
Crude fiber (%)^3)^	5.50	6.10	9.80	8.70	7.00
Crude protein (%)^3)^	20.81	20.80	20.81	20.81	20.80
Total calcium (%)^3)^	0.83	0.84	0.84	0.83	0.83
Total phosphorus (%)^3)^	0.63	0.72	0.76	0.71	0.67
Total lysine (%)^3)^	1.25	1.29	1.31	1.29	1.27
Total methionine (%)^3)^	0.62	0.61	0.60	0.60	0.62

1) The premix provided the following amounts: Vit A 28,500 IU; Vit D_3_ 6,000 IU; Vit E 67.5 IU; Vit K_3_ 7.5 mg; Vit B_1_ 7.5 mg; Vit B_2_ 15 mg; Vit B_6_ 9 mg; Vit B_12_ 0.075 mg; niacin 75 mg; calcium pantothenate 37.5 mg; folic acid 3 mg; biotin 0.375 mg; choline chloride 100 mg; antioxidant 0.15 mg; Fe 150 mg; Cu 200 mg; I 0.4 mg; Se 0.3 mg; Zn 300 mg; Co 1 mg; Mn 60 mg.

2) Calculated.

3) Analyzed.

**Table 3:** Dietary starch digestibility in small intestine of weaned piglets in Exp 1

Items	Treatment			

	Diet I	Diet II	Diet III	Diet IV
NR_S_ (%)	25.80±0.59^a^	31.67±0.23^b^	45.71±1.65^c^	49.36±1.00^d^
NF_S_ (%)	12.38±2.27^a^	13.21±2.00^ab^	13.48±2.58^ab^	16.30±0.76^b^
IR_T_ (g/kg)	1.27±0.13	1.41±0.19	1.38±0.04	1.19±0.25
IF_T_ (g/kg)	3.38±0.54	3.74±0.59	4.07±0.48	3.54±0.63
D_DM_ (%)	64.98±5.68^ab^	62.10±6.49^a^	66.33±4.78^ab^	72.00±1.68^b^
D_S_ (%)	81.73±6.85^a^	82.40±5.32^a^	89.89±2.25^b^	90.32±0.08^b^

NR_S_, starch contents in feed; NF_S_, starch contents in small intestine digesta; IR_T_, titanium dioxide contents in diet; IF_T_, titanium dioxide in contents in small intestine digesta; D_DM_, the small intestine digestibility of dry matter; D_S_, the small intestine digestibility of starch.

a–d Means within a row with different superscripts differ significantly (p<0.05).

**Table 4:** Lactic acid and propionic acid net absorption in the portal vein of weaned piglets in Exp 1

Items	Treatment			

	Diet I	Diet II	Diet III	Diet IV
F (L/kg/h)	4.85±0.48^a^	9.98±0.96^c^	6.32±0.2^ab^	8.18±1.44^bc^
C_pa_ (μg/mL)^1)^	ND	ND	ND	ND
P_pa_ (μg/mL)	2.50±0.24	3.21±0.48	3.49±1.11	3.34±1.17
C_la_ (g/L)	0.19±0.02^b^	0.17±0.01^ab^	0.16±0.03^a^	0.17±0.01^ab^
P_la_ (g/L)	0.21±0.02^b^	0.17±0.01^a^	0.17±0.02^a^	0.17±0.003^a^
F_pa_ (g/d)	2.22±0.34^a^	6.01±0.23^b^	3.87±1.35^ab^	3.50±0.94^a^
F_la_ (g/d)	13.91±3.77^b^	8.87±1.68^ab^	6.28±2.35^a^	3.97±1.22^a^

F, the portal blood flow rate; C_pa_, P_pa_, the concentrations of propionic acid in carotid artery and portal plasma, respectively; C_la_, P_la_, the concentrations of lactic acid in carotid artery and portal plasma, respectively; F_pa_, F_la_, the propionic acid and lactic acid net absorption of the portal vein, respectively; ND, not detected.

1) The propionic acid was not detected in carotid artery.

a–c Means within a row with different superscripts differ significantly (p<0.05).

**Table 5:** The metabolizable glucose levels of diets for weaned piglets in Exp 1

Items	Treatment			

	Diet I	Diet II	Diet III	Diet IV
T_IDSG_ (g/d)	53.15±4.45^a^	65.88±4.25^b^	103.54±2.60^c^	111.66±0.25^d^
D_IDSG_ (g/kg)	189.82±15.90^a^	235.30±15.19^b^	369.80±9.29^c^	398.79±0.88^d^
T_LAFG_ (g/d)	13.94±2.99	12.71±1.65	8.68±0.92	15.56±4.03
D_LAFG_ (g/kg)	49.80±10.68	45.38±5.88	30.99±3.29	55.56±14.39
T_MG_ (g/d)	67.09±7.90^a^	78.59±4.90^a^	112.22±1.51^b^	127.22±3.78^c^
D_MG_ (g/kg)	239.62±28.22^a^	280.68±17.52^a^	400.79±5.38^b^	454.35±13.51^c^

T_IDSG_, D_IDSG_, the exogenous metabolizable glucose intake and the exogenous metabolizable glucose levels in the diet, respectively; T_LAFG_, D_LAFG_, the endogenous metabolizable glucose intake and the endogenous metabolizable glucose levels in the diet, respectively; T_MG_, D_MG_, the metabolizable glucose intake and the metabolizable glucose levels in the diet, respectively.

a–d Means within a row with different superscripts differ significantly (p<0.05).

**Table 6:** The regression equation of three metabolizable glucose levels in diets and feedstuffs in Exp 1

Items	Regression equation	Statistical parameter	

		R^2^ ^2)^	p-value
IDSG	Y_1_ = −110.82+12.07 X_1_+5.80X_2_	0.9995	0.030
LAFG	Y_2_ = −106.70+2.35X_1_+18.48X_2_	0.6298	0.608
MG	Y_3_ = −196.44+12.13X_1_+23.18X_2_	0.9989	0.033

IDSG, exogenous metabolizable glucose; LAFG, endogenous metabolizable glucose; MG, metabolizable glucose.

1) Y_1_, the exogenous metabolizable glucose levels in diet; Y_2_, the endogenous metabolizable glucose levels in diet; Y_3_, the metabolizable glucose levels in diet; X_1_, the starch content of diet; X_2_, the crude fibre content of diet.

2) The goodness of fit of regression equation.

**Table 7:** Effects of different levels metabolizable glucose on growth performance and nutrients digestibility in piglets in Exp 2

Item	Treatment				

	Diet A	Diet B	Diet C	Diet D	Diet E
Growth performance					
ADFI (g/d)	471±43^a^	665±63^b^	665±27^b^	703±54^bc^	782±53^c^
ADG (kg/d)	0.23±0.05^a^	0.51±0.06^c^	0.49±0.11^c^	0.28±0.10^ab^	0.38±0.13^b^
FCE (%)	0.49±0.09^a^	0.78±0.07^b^	0.73±0.15^b^	0.41±0.12^a^	0.53±0.10^a^
Digestibility of nutrients					
TD_DM_ (%)	55.36±9.56^a^	65.72±4.67^b^	63.45±8.07^ab^	57.05±10.82^ab^	75.75±2.11^c^
TD_CP_ (%)	33.67±10.18^a^	67.36±6.90^b^	71.25±5.09^b^	69.13±11.87^b^	67.99±7.68^b^
TD_CF_ (%)	86.14±5.14^b^	84.26±5.74^b^	82.07±4.96^b^	73.86±9.07^a^	80.48±3.81^ab^

ADFI, average daily feed intake; ADG, average daily gain; FCE, feed conversion efficiency; TD_DM_, the total digestive tract digestibility of dry matter; TD_CP_, the total digestive tract digestibility of crude protein; TD_CF_, the total digestive tract digestibility of crude fat.

a–c Means within a row with different superscripts differ significantly (p<0.05).
