# Supplementary material for: A regression for estimating metabolizable glucose in diets of weaned piglets for optimal growth performance
Source: Anim Biosci. 2020 Dec 11;34(10):1643–52. doi: 10.5713/ab.20.0459 (PMC8495354; doi:10.5713/ab.20.0459)
Supplement: Supplementary file 1 [file ab-20-0459-suppl.pdf]

S-Table 1

Composition of the diets (%, as-fed basis) and nutritional composition in Exp. 2

Items	Treatment				
	Diet V	Diet VI	Diet VII	Diet VIII	Diet IX
Ingredient (%) [1)]					
Corn	0	5.54	16.1	34.6	19.3
Corn starch	0	0	0	0	19
Whey powder	9.6	8.87	8.31	8	8
Wheat bran	0	11.09	12.46	1	0
Rice chaff	5.21	2.99	2.21	3.18	3.8
Soybean oil	25.45	20.07	15.02	9.86	7.71
Soybean meal	12.01	11.09	10.39	10	10
Extruded soybean	12.01	11.09	10.39	10	10
Fish meal	4.8	4.44	4.15	4	4
Plasma protein meal	3.6	3.33	3.12	3	3
Corn gluten meal	0.6	0.55	0.52	0.5	0.5
Limestone	1.44	1.33	1.25	1.2	1.2
Calcium hydrophosphate	0.36	0.33	0.31	0.3	0.3
Salt	3	2.77	2.6	2.5	2.5
Premix [2)]	6	5.54	5.19	4.31	0.61
Zeolite powder	15.27	10.40	7.46	7.05	9.55
Lysine	0.24	0.22	0.21	0.2	0.2
Methionine	0.28	0.22	0.23	0.2	0.23
Titanium dioxide	0.1	0.1	0.1	0.1	0.1
Total	100	100	100	100	100
Nutritional composition					
Metabolizable glucose (g/kg) [3)]	37.6	132.5	300.0	354.3	412.5
Metabolic energy (MJ/kg) [3)]	13.67	13.67	13.68	13.66	13.65
Digestive energy (MJ/kg) [3)]	14.68	14.67	14.68	14.68	14.68
Crude protein (%) [4)]	20.81	20.80	20.81	20.81	20.80
Starch (%) [4)]	8.7	15.5	22.2	28.7	36.7
Crude fiber (%) [4)]	5.5	6.1	9.8	8.7	7.0
Total calcium (%) [4)]	0.83	0.84	0.84	0.83	0.83
Total phosphorus (%) [4)]	0.63	0.72	0.76	0.71	0.67
Total Lysine (%) [4)]	1.25	1.29	1.31	1.29	1.27
Total Methionine + cystine (%) [4)]	0.91	0.89	0.92	0.91	0.92

[1)] The nutrient content of each feed in the formula is calculated according to the NRC (2012), Extra add Titanium dioxide (1 g per kg diet) as the indigestible marker to measure nutrient digestibility.

[2)] The premix provided the following amounts: VA 28,500 IU ; VD3 6,000 IU; VE 67.5 IU; VK3 7.5 mg; VB1 7.5 mg; VB2 15 mg; VB6 9 mg; VB12 0.075 mg; niacin 75 mg; calcium pantothenate 37.5 mg; folic acid 3 mg; biotin 0.375 mg; choline chloride 100 mg; antioxidant 0.15 mg; Fe 150 mg; Cu 200 mg; I 0.4 mg; Se 0.3 mg; Zn 300 mg; Co 1 mg; Mn 60 mg;.

[3] Calculated.

[4] Analyzed.
